# Sediment transport modeling in non-deposition with clean bed condition using different tree-based algorithms

**DOI:** 10.1371/journal.pone.0258125

**Published:** 2021-10-08

**Authors:** Enes Gul, Mir Jafar Sadegh Safari, Ali Torabi Haghighi, Ali Danandeh Mehr

**Affiliations:** 1 Department of Civil Engineering, Inonu University, Malatya, Turkey; 2 Department of Civil Engineering, Yaşar University, Izmir, Turkey; 3 Water, Energy and Environmental Engineering Research Unit, University of Oulu, Oulu, Finland; 4 Department of Civil Engineering, Antalya Bilim University, Antalya, Turkey; Ton Duc Thang University, VIET NAM

## Abstract

To reduce the problem of sedimentation in open channels, calculating flow velocity is critical. Undesirable operating costs arise due to sedimentation problems. To overcome these problems, the development of machine learning based models may provide reliable results. Recently, numerous studies have been conducted to model sediment transport in non-deposition condition however, the main deficiency of the existing studies is utilization of a limited range of data in model development. To tackle this drawback, six data sets with wide ranges of pipe size, volumetric sediment concentration, channel bed slope, sediment size and flow depth are used for the model development in this study. Moreover, two tree-based algorithms, namely M5 rule tree (M5RT) and M5 regression tree (M5RGT) are implemented, and results are compared to the traditional regression equations available in the literature. The results show that machine learning approaches outperform traditional regression models. The tree-based algorithms, M5RT and M5RGT, provided satisfactory results in contrast to their regression-based alternatives with *RMSE* = 1.184 and *RMSE* = 1.071, respectively. In order to recommend a practical solution, the tree structure algorithms are supplied to compute sediment transport in an open channel flow.

## 1. Introduction

For the hydraulic design of urban drainage systems, the sediment transport process must be considered. Flow, fluid, sediment, and channel characteristics related to the sedimentation issues should be considered in order to design wastewater and sewage pipes [1, 2]. Channels are designed to minimize the deposition of sediment depending on different success criteria. Self-cleansing is a concept used in channel construction to encompass non-deposition requirements [3]. Non-deposition involves three criteria: incipient deposition (ID), non-deposition with a clean bed (NCB) and non-deposition with a deposited bed (NDB). The NCB criterion can be implemented by adapting the flow shear stress or velocity to satisfy the clean bed condition [4, 5]. ID is the moment when suspended particles begin to settle. In addition, in NDB criterion, an appropriate deposited bed depth is used to decrease the channel building costs [6, 7].

The non-deposition sediment transport equations were recommended to ensure the clean bed criterion by adopting the required velocity or sediment concentration, as shown in Table 1. The study conducted by [8] documented how the size of the granular material has a substantial effect on the transportation volume of sediment. The non-deposition condition in suspended sediment transport has already been examined by [9–11]. Comprehensive experiments on bed load sediment transport showed that the design velocity is increased as the pipe dimension is expanded [12]. The utility of previously recommended sewer design formulas was evaluated by [13–15]. [16] examined the Camp approach to sewer design and showed that the flow velocity was greatly overestimated. Experiments were carried out in a large pipe, and new self-cleansing models were recommended by [17]. [2] utilized [18] experimental data conducted in five different cross-section channels of rectangular, trapezoidal, V-bottom and U-shape and developed a self-cleansing model considering channel cross-section shape.

**Table 1:** Traditional regression equations in literature for the sediment transport self-cleansing condition.

Model		Reference	Eq. No
$\frac{V}{\sqrt{gd(s-1)}}=$	$3.08C_{v}^{0.21}D_{gr}^{-0.09}{\left(R/d\right)}^{0.53}\lambda^{-0.21}$	[12]	(1)
	$4.31C_{v}^{0.226}{\left(d/R\right)}^{-0.616}$	[16]	(2)
	$4.79C_{v}^{0.209}{\left(d/R\right)}^{-0.593}\lambda^{0.058}$	[17]	(3)
	$4.344C_{v}^{0.181}Dgr^{-0.088}{\left(d/R\right)}^{-0.48}\lambda^{-0.092}$	[34]	(4)
	$4.83C_{v}^{0.09}D_{gr}^{-0.14}{\left(d/R\right)}^{-0.32}{\left(P/B\right)}^{0.2}$	[2]	(5)

*D*_*gr*_: dimensionless grain size (–); *P*: wetted perimeter (m); *B*: water surface width (m); *d*: sediment median size (m); *λ*: channel friction factor (-); *R*: hydraulic radius (m); *V*: flow mean velocity (m s^-1^); *C*_*v*_: sediment volumetric concentration (-); *s*: relative specific mass of sediment to fluid (–) and *g*: gravitational acceleration (m s^−2^).

Owing to the robustness of machine learning algorithms, their application on sediment transport modeling has attracted great interest in the literature [3, 19–22]. Various machine learning algorithms have recently been suggested for modeling open channel sediment transport over conventional regression equations. However, utilizing a limited data range for model development is the main limitation of previous studies. For example, different algorithms were applied to model sediment transport in an open channel, such as classification-based [23–25], tree-based [26], network-based [27–29] and evolutionary algorithms [30–33].

It is known that experimental data range, implemented machine learning algorithm and using effective parameters for model development based on the physics of the problem, are quite essential factors to construct a robust machine learning model. As an extension of the existing studies in the literature, in order to promote the modeling of sediment transport in non-deposition with clean bed condition of sediment transport all aforesaid factors are considered in this study. The improved M5 rule tree (M5RT) and M5 regression tree (M5RGT) have been used for solving numerous engineering problems [35, 36]. M5RT and M5RGT are useful for generating a rules-based and fingerprint models from a data set. Thus, in this study, tree-based algorithms of improved M5RT and M5RGT are used to model sediment transport in open channels. While majority of studies in the literature utilized limited amount of data for modeling, this study utilized six data sets having wide ranges of channel size and shape, sediment median size and volumetric concentration, channel bed slope and flow depth. Relying on the hydraulics of sediment transport, models are developed through considering fluid, flow, channel, and sediment characteristics.

## 2. Methodology

### 2.1. Data preparation

Experimental studies reported for NCB condition taken from the literature are used in this study. The large data set used in this study was compiled from [12, 16–18, 37, 38]. [37] performed experiments in two cross-sectional shapes, circular and rectangular. The tests were conducted with six different sizes of granular materials in a range between 0.5 to 8.74 mm. In the experiments [38] conducted tests in a circular pipe using granular material with a size of 0.73 mm. [12] conducted experiments with circular cross-sectional shapes, with granular material sizes ranging from 0.46–8.3 mm utilized in the experiments. [16] performed experiments in circular cross-sectional shape with two channels and three different granular materials with sizes between 0.2–0.43 mm. For more detail on this experimental data, the interested reader may refer to [29, 39].

As a novel contribution, the data of [17 and 18] are included in this study. The data used in these two studies enhances the modeling reliability. Thus, the use of data from [18] makes this study more reliable in obtaining experimental data with a variety of cross-sectional shapes, with experiments performed in trapezoidal, circular, U-shape, rectangular and V-shape bottom channels. Also [17], who used a large-diameter (595 mm) circular cross-sectional channel, makes this study more reliable than the models in the literature. The ranges of data sources are given in Table 2.

**Table 2:** Range of data used in the present study.

	*C*_*v*_ *(ppm)*	*D*_*gr*_	*d/R*	*λ*	*Fr*_*p*_
[37]	14.2–1568	12.6–221.8	0.006–0.41	0.014–0.03	1.3–10.8
[38]	2.3–22.1	18.46	0.005–0.0065	0.0142–0.0182	4.6–9.9
[12]	4–1450	11.49–205.62	0.005–0.259	0.0129–0.0475	1.29–13.53
[16]	4–90	5.05–10.88	0.007–0.0252	0.038–0.0531	2.86–8.99
[18]	112–9814	3.75–38.05	0.004–0.1416	0.0198–0.1651	1.56–12.81
[17]	1–19957	8.85–65.63	0.0033–0.225	0.0036–0.0742	2.88–16.07

It is demonstrated by [1, 2] that four characteristics of fluid, flow, channel, and sediment must be embedded to a sediment transport model. As already reported in the literature, self-cleansing models can be influenced by the following parameters;

$$f(V,g,\rho ,\upsilon ,R,d,\lambda ,C_{v},\rho_{s})=0$$ (6)

where *V* is flow velocity, *g* is gravity acceleration, *ρ* is fluid specific mass, *υ* is fluid kinematic viscosity, *R* is hydraulic radius, *d* is median size of sediment, *λ* is channel friction factor, *C*_*v*_ is sediment volumetric concentration, and *ρ*_*s*_ is sediment specific mass. These parameters can be considered effective sediment transport variables for the modeling. The following equation is written taking the above variables into consideration as a group of dimensionless parameters:

$$Fr_{p}=f(C_{v},D_{gr},d/R,\lambda )$$ (7)

where *D*_*gr*_ and *Fr*_*p*_ are the dimensionless grain size and particle Froude number parameter, respectively defined as:

$$D_{gr}={\left(\frac{(s-1)gd^{3}}{v^{2}}\right)}^{1/3}$$ (8)

$$Fr_{p}=(V/\sqrt{gd(s-1}))$$ (9)

where *s* is relative particle mass (*s* = *ρ*_*s*_/*ρ*). Table 3 shows statistical characteristics of the utilized data. In this study, the data set was divided into 70% training and 30% testing data. Based on the findings published by [40], who performed uncertainty analysis, the split between training and test data does not make a major difference to model performance; however, the best data split rate was reported as 70% and 30% for training and testing periods.

**Table 3:** Statistical characteristics of the data used for modeling.

	Range	Mean	Std. Deviation	Skewness	Kurtosis
*C*_*v* (ppm)_	2–0.0016	0.0285	0.000326	1.7864	5.6288
*D*_*gr*_	5.05–215.59	65.1812	59.5500	1.2067	3.4910
*d/R*	0.0052–0.4162	0.0671	0.0751	1.9584	7.2243
λ	0.0107–0.0532	0.0215	0.0085	1.5197	5.0678
*Fr*_*p*_	1.2984–13.5292	4.4056	2.2609	0.9741	3.8024

### 2.2. M5P classifiers

#### 2.2.1. M5 regression tree

The M5 Model Trees are a state-of-the-art algorithm that effectively divides the sample area into subspaces and forms linear regression models in pieces for each subspace [41]. Model trees are a more general form of regression tree, with constant values as their leaves [42]. M5’, also known as M5P, is an improved version of the Classification and Regression Tree (CART). A task was done to reduce the number of trees in the original M5P algorithm. It differs from other tree-based solutions due to the use of linear functions in the leaves. The linear function used at the decision tree nodes divides the tree into leaves to form the model tree. Tree-based models are constructed using a divide and conquer method.

The model tree is created in three stages. The first stage involves branching according to the splitting criterion. The branching criterion depends on the value of the standard deviation of the attribute value. The attribute that reduces the expected error is chosen as the root of the tree. The formula for standard deviation reduction (*SDR*) is calculated as in Eqs (10–12).

$$SDR=sd(T)-\sum_{i}\frac{\left|T_{i}\right|}{T}sd(T_{i})$$ (10)

$$sd(T)=\sqrt{\frac{1}{n-1}}\sum_{i=1}^{n}{\left(T_{i}-\bar{T}\right)}^{2}$$ (11)

$$\bar{T}=\frac{1}{n}\sum_{i=1}^{n}T_{i}$$ (12)

where *n* is the number of training examples at the node, *T* is a set of attributes that reaches the node, $\bar{T}$ is the average value of the sets of *T* attribute and *sd*(*T*) is the standard deviation of *T*.

The second stage is tree pruning. Each leafless node of the model tree is examined, starting from below, for the pruning stage, as in Eq (13). The M5 algorithm selects the final model for this node of simplified linear model or model subtree, depending on the minimized estimated error rate. The final stage is tree smoothing.

$$\frac{n+v}{n-v}$$ (13)

where *n* is the number of training examples at the node and *v* is the number of parameters representing class value at the node.

**Fig 1:**
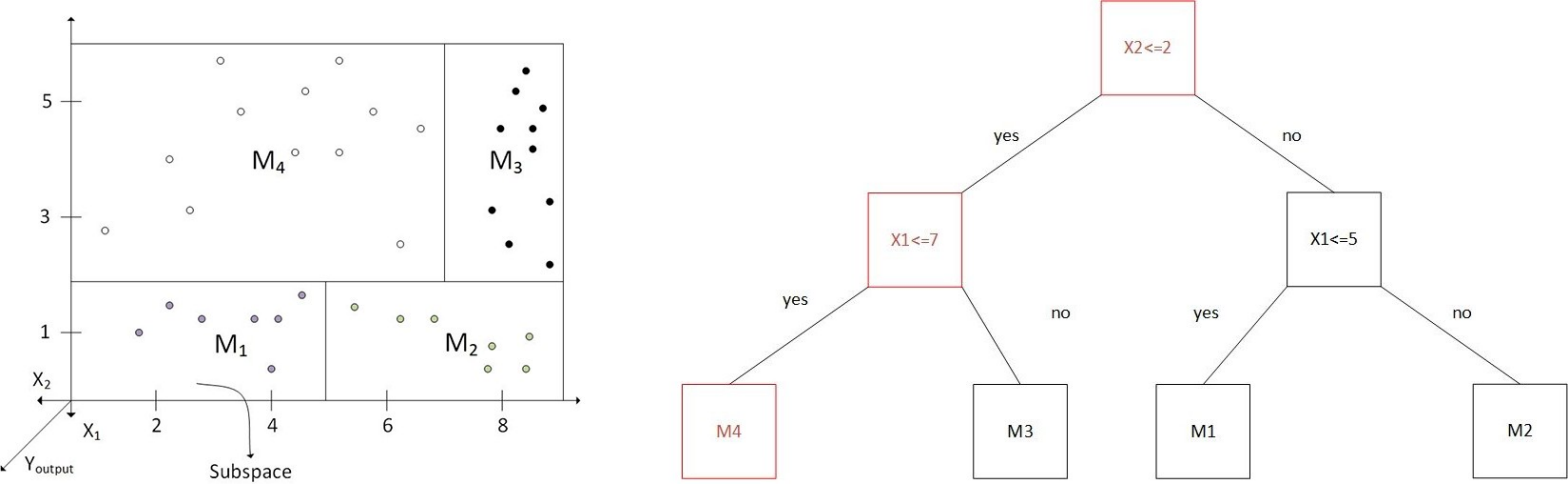
Schematic for the M5P method.

The difference between real class value and predicted value is averaged in every training example to reach the node for the pruning process in the M5 algorithm. This average value is multiplied by this coefficient. The M5 Regression tree operates in the same way as M5P model tree in all steps. The value of subspaces that act as dividers is not a linear equation in the RGT model (Fig 1).

#### 2.2.2. M5 rule tree

M5 rule generates the rules from the M5 tree, based on the CART algorithm that was presented by [43]. The method for generating rules from model trees, which we call M5’ Rules, works as follows: a tree learner (in this case model trees) is applied to the whole training data and a pruned tree is developed. Next, the best leaf is made into a rule. All samples covered by the rule are removed from the dataset. The process is applied recursively to the remaining samples and terminates when all samples are covered by one or more rules. In contrast to CART, which employs the same strategy for categorical prediction, M5’ Rules builds full trees, instead of partially explored trees. All modeling was done in a Matlab 2016B environment [44]. Table 4 shows model parameters for M5RGT and M5RT.

**Table 4:** Model parameters for M5RGT and M5RT.

	M5RGT-M5RT
Minimum leaf size	2
Minimum parent size	4
Split threshold	5%
Maximum depth	No limitation

### 2.3. Performance criteria

The analysis of model performance is important for investigating the credibility of the models. Thus, the coefficient of determination (*R*^*2*^), variance account for (*VAF*), root mean square error (*RMSE*), Nash–Sutcliffe model efficiency coefficient (*NASH*), and *a*10-index are used in this study to determine the degree of fit index. The model performs well with *R*^*2*^, *NASH* and *a*10-*index* close to the unity, *VAF* close to 100 and *RMSE* close to zero. The *R*^*2*^, *VAF*, *RMSE*, *NASH* and *a*10-*index* can be computed using the following equations, respectively:

$$R^{2}={\left(\frac{\sum_{j=1}^{m}\left(p_{j}-p_{j,t}\right)\left({\overset{⌢}{p}}_{j}-{\overset{⌢}{p}}_{j,t}\right)}{\sqrt{\sum_{j=1}^{m}{\left(p_{j}-p_{j,t}\right)}^{2}\sum_{j=1}^{m}{\left({\overset{⌢}{p}}_{j}-{\overset{⌢}{p}}_{j,t}\right)}^{2}}}\right)}^{2}$$ (14)

$$VAF=(1-\frac{\mathrm{variance}(p_{j}-{\overset{⌢}{p}}_{j})}{\mathrm{variance}(p_{j})})100$$ (15)

$$RMSE=\sqrt{\frac{1}{m}\sum_{j=1}^{m}{\left(p_{j}-{\overset{⌢}{p}}_{j}\right)}^{2}}$$ (16)

$$NASH=1-[\frac{\frac{1}{m}\sum_{j=1}^{m}{\left(p_{j}-{\overset{⌢}{p}}_{j}\right)}^{2}}{\frac{1}{m}\sum_{j=1}^{m}{\left({\overset{⌢}{p}}_{j}-p_{j,t}\right)}^{2}}]$$ (17)

$$a10-index=\frac{k10}{K}$$ (18)

where, *p*_j_ is the observed value, ${\overset{⌢}{p}}_{j}$ is the predicted value, subscript of *t* indicates the mean value, *m* is the data number, *K* is the total number in the dataset, and *k*10 is the number of samples in the case with a ratio of measured values to predicted values between 10% error (0.9 < observed/predicted < 1.1). Each performance criterion examines specific feature of the developed model as *R*^*2*^ shows the correlation, *RMSE* scattering rates, *VAF* variance variation, *NASH* data skewness and *a*10-*index* gives information about model reliability.

## 3. Results

The performances of two tree-based algorithms, M5RT and M5RGT, are compared by means of the different statistical error measurement criteria of *R*^*2*^, *VAF*, *RMSE*, *NASH* and *a*10-*index* in Table 5. Performance of M5RT and M5RGT models recommended in this study are examined in comparison to four empirical equations of [2, 12, 16, 17, 34]. [12 and 16] equations are selected due to their credibility as reported in [39]. Furthermore, recently reported equations of [2, 17, 34] are used for model performance evaluation. The results showed that M5RGT is slightly superior to M5RT, in terms of different statistical performance criteria. Both models proposed in this study performed better than traditional equations. Among regression models, [16] generates the poorest results, while [2] provide better results in contrast to other regression equations. A comparison of best machine leaning models of M5RGT with *RMSE* of 1.071 with the best regression equation of [2] with *RMSE* of 1.350 illustrates an almost 21% improvement on the accuracy of the model in M5RGT algorithm.

**Table 5:** Model performances with different statistical indexes.

	*R* ^2^	*VAF*	*RMSE*	*NASH*	*a*10-*index*
Train					
M5RT	0.934	93.407	0.670	0.929	0.716
M5RGT	0.960	95.955	0.525	0.958	0.808
Test					
[12]	0.801	76.876	2.003	0.495	0.3812
[16]	0.785	74.508	2.201	0.680	0.3481
[17]	0.779	76.219	1.842	0.551	0.3978
[34]	0.812	80.608	1.559	0.678	0.3978
[2]	0.782	62.093	1.350	0.752	0.3646
M5RT	0.814	80.456	1.184	0.804	0.4475
M5RGT	0.842	81.580	1.071	0.840	0.3923

Over-fitting and under-fitting are of great importance in determining the accuracy of the model. Over-fitting means the model memorizes while training. Therefore, test performance is significantly worse than training performance. On the other hand, in the case of under-fitting, test results are better than training results. The best-case scenario is the model performance in the training and testing phases being close to each other. Table 5 shows how the results of four different models in the training and testing phases have no significant differences, so it can be said that the developed models in this study have no such deficiencies.

**Fig 2:**
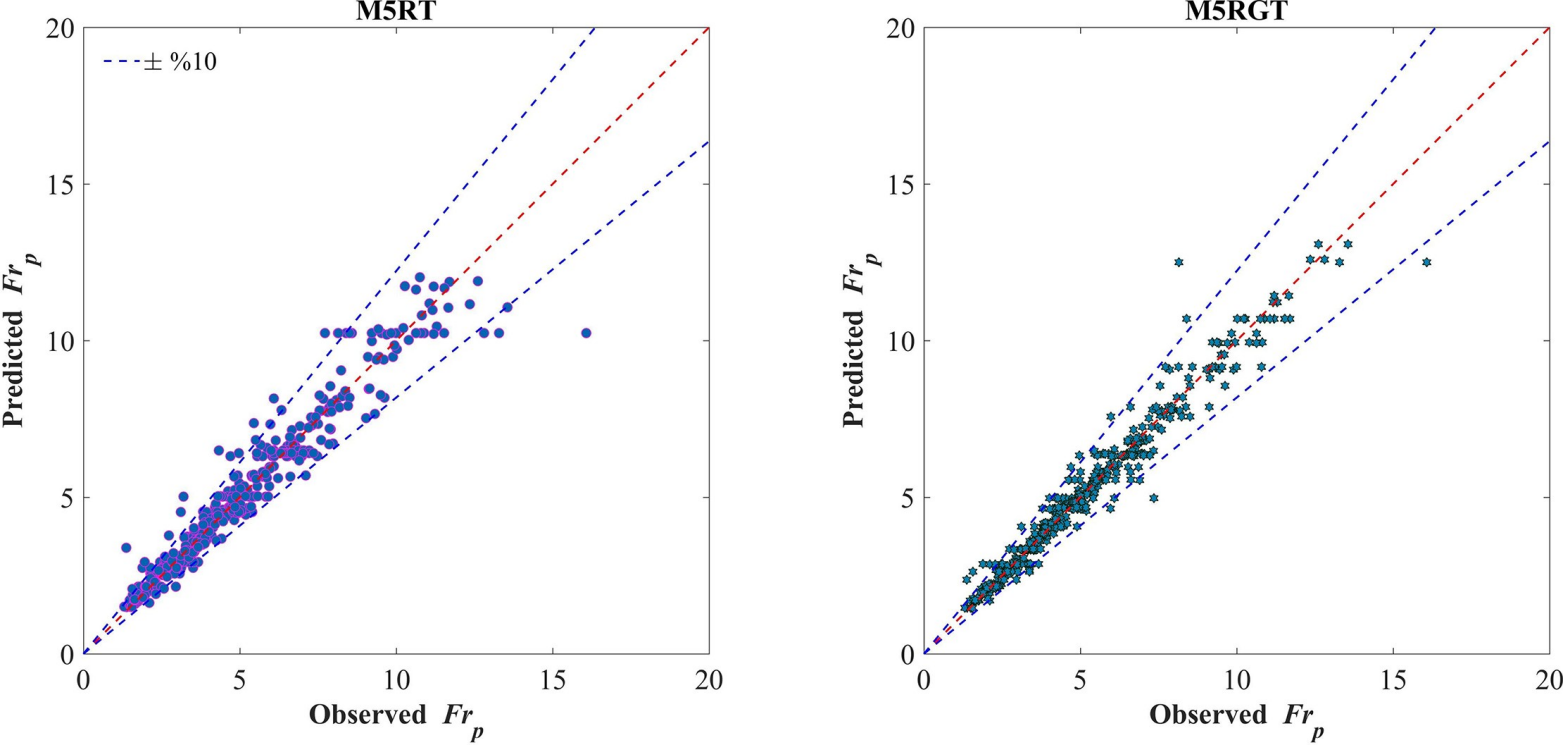
Scatter plots of observed and predicted particle Froude numbers for the training stage.

Fig 2 shows the scatterplot of the observed and predicted values for different models during the training phase. These are two major problems for the underestimation or overestimation of sediment transport models. If the predicted value is greater than the observed value, an overestimation problem occurs; otherwise, if predicted values are lower than the actual counterparts, underestimation will occur. An overestimated model could not be an economical design method as it causes open channels to be designed larger than required. An underestimated model, on the other hand, causes the channel to be designed without sufficient planning criteria. As shown in Fig 2, bisector lines for both models are passed through the middle of the data clouds showing that developed models in this study have no significant over- or underestimation.

**Fig 3:**
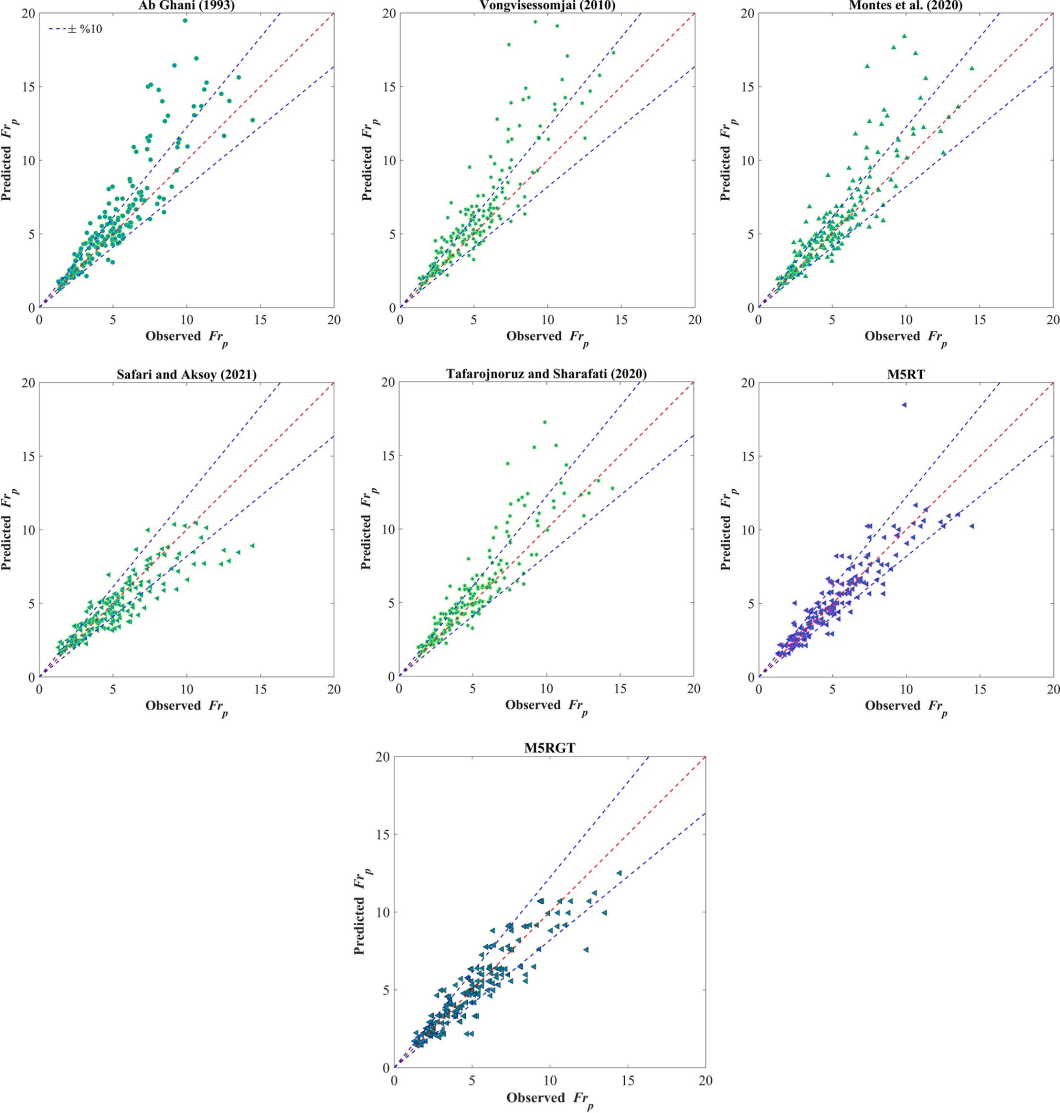
Scatter plots for observed and predicted particle Froude numbers for the testing stage.

The visually comparison of M5RT and M5RGT with conventional equations in the form of scatter plots are shown in Fig 3. It can be seen from Fig 3 that M5RT and M5RGT outcomes are close to the best fit line, showing their accuracy where, except a few data points, most of the data falls within the ±10% lines. In the case of regression equations, a significant scatter has been seen, showing their deficiency in accurate sediment transport prediction. The equations of [12, 16, 17] generate considerable overestimation, while the equation of [2] shows a slight underestimation.

## 4. Discussions

As noted earlier, machine learning models for sediment transport are superior to traditional regression equations. While traditional regression equations are more practical than machine learning methods, they have not produced reliable models. For this reason, model trees methods with practical application areas were proposed as an alternative to classical regression equations in this study (their codes are given as S1 Table).

The data range is quite important for the reliability of the model for sediment transport in open channels. In this study, a more comprehensive data set than the data used in studies found in the literature is used for the first time. The wide range of data makes it difficult to establish an accurate and reliable model. The methods (M5RT and M5RGT) used in this study performed better than the methods recommended in the literature, although the utilized data has a considerable wide range. This study examines the performance of traditional equations when using this large data set. According to the traditional regression equations’ performances, the [2] equation was found to have the best performance in contrast to the other equations. Modeling such a large data set can reveal the problem of overestimation and underestimation. In particular, it was observed that the traditional regression equations overestimated, except for the [2] equation, which has a slight underestimation. Additionally, it was found that traditional equations are overestimated models for analyzing sediment transport in self-cleansing design using limited data sets [29, 45].

In this study, a new index, *a*10-*index*, is also used. The *a*10-*index* shows the amount of data between the 10th percentiles. Examining the *a*10-*index* performances of the models, together with the *RMSE*, enabled more reliable models to be obtained. Examining such a large data set is important in terms of observing the outlier effect. M5RT was superior to M5RGT in terms of *a*10-*index* performance criterion. As can be understood from the results obtained in this study, the outlier performance is found to be better in M5RGT model.

It must be noticed that over-fitting is an essential issue in application of machine learning algorithms [29, 46]. Incorporating a few number of input variables and weights to construct a well-organized model works out over-fitting issue [47]. An over-fitted model fits on train data set perfectly and provides weak results on test data set [48]. The recommended models in this study for sediment transport modeling are not expected to encounter with over-fitting issue as developed models perform well on test data set. Most importantly, the developed models in this study are constructed on large data sets and model parameters are elected relying on the physics of the sediment transport in open channel flow.

The results obtained in this study illustrate that the recommended M5RT and M5RGT models give better outcomes in comparison to the empirical equations of [2, 12, 16, 17]. Implementation of robust machine leaning algorithms for solving complex and difficult hydraulic problems such as sediment transport is inevitable. However, considering effective variables based on the physics of the problem, and experimental data range are of prominent importance to get reliable results. While most of the studies in the literature used a few data sets for modeling sediment transport at non-deposition with clean bed condition, this study extends the available studies in the literature through utilizing wider range of experimental data taken from six sources which cover wide ranges of channel size and shape, sediment median size and concentration and flow depth. As a result, it seems that this study favorably developed sediment transport models which can be used in practice for channel design purpose.

In this study, the laboratory data collected from the literature was used for sediment transport modeling. The authors acknowledge that more reliable modeling could be done using the field data obtained in practice. Although tree-based algorithms produce solutions that can be used in practice, using alternative methods, such as symbolic regression or bagged tree, is recommended in future studies. In addition, a detailed examination of the outlier effect will make important contributions to future studies.

## 5. Conclusions

In this study, modeling of the sediment transport in open channels is conducted using two different tree-based algorithms, M5 rule tree (M5RT) and M5 regression tree (M5RGT). The six existing data sets with a wide range compiled from the literature are used for model development. The proposed algorithms, M5RT and M5RGT, are compared to traditional regression equations, in particle Froude number prediction. Our results indicated that the M5RGT outperforms M5RT with *RMSE* = 1.071 and *RMSE* = 1.184, respectively. Modeling results show that the proposed algorithms are superior to the traditional equations. According to traditional regression equations’ performances, most of the models show significant overestimation, demonstrating their deficiency in terms of economical design benchmarks, where channels need steeper bed slope to satisfy the non-deposition sediment transport condition. In addition, a new index, *a*10-index, is presented in this study to enhance the model performance examination. The tree structures are presented explicitly and are expected to provide practical solutions for users. Future research directions can be considered based on the limitation of this study in terms of utilized data for the modeling. Conducting filed studies to collect data from real sewers and drainage systems through incorporating cohesive sediment particle characteristics may improve the credibility of the developed models.

## Supporting information

S1 Table M5 regression tree (M5RGT) structure. (DOCX)
