# Supplementary material for: Sediment transport modeling in non-deposition with clean bed condition using different tree-based algorithms
Source: PLoS One. 2021 Oct 8;16(10):e0258125. doi: 10.1371/journal.pone.0258125 (PMC8500418; doi:10.1371/journal.pone.0258125)
Supplement: S1 Table — (DOCX) [file pone.0258125.s001.docx]

**Supporting information**

**Table S1**. M5 regression tree (M5RGT) structure

| Number of rules: 115  Number of original input variables used: x1 (*C_v_*), x2 (*D_gr_*), x3 (*d/R*) and x4 (*λ*)  The tree:  if x2 <= 24.361  if x2 <= 9.8654  if x1 <= 8.4e-05  if x1 <= 1.55e-05  y = 4.5633 (3)  else  if x3 <= 0.013889  if x2 <= 6.324  y = 7.7633 (3)  else  y = 6.4824 (3)  else  y = 5.56 (3)  else  if x4 <= 0.076089  if x4 <= 0.06631  if x4 <= 0.043532  if x4 <= 0.016904  if x3 <= 0.005904  y = 10.23 (2)  else  y = 7.5729 (3)  else  if x4 <= 0.022144  y = 12.584 (2)  else  if x1 <= 0.003717  if x1 <= 0.0013871  y = 10.69 (11)  else  if x1 <= 0.002275  y = 13.079 (2)  else  y = 11.227 (2)  else  y = 9.9201 (3)  else  if x4 <= 0.060869  y = 9.1609 (8)  else  y = 7.5303 (2)  else  y = 12.503 (3)  else  y = 5.8065 (2)  else  if x4 <= 0.045347  if x1 <= 0.00062341  if x1 <= 1.345e-05  if x3 <= 0.0096476  if x1 <= 4.35e-06  if x4 <= 0.0154  y = 5.3486 (2)  else  y = 4.6835 (3)  else  if x4 <= 0.01685  if x3 <= 0.0062554  y = 6.3945 (3)  else  y = 7.2441 (3)  else  y = 5.9615 (6)  else  y = 4.1618 (2)  else  if x3 <= 0.010565  if x4 <= 0.02943  if x3 <= 0.0067981  if x1 <= 2.025e-05  y = 7.879 (7)  else  y = 9.0672 (5)  else  if x1 <= 7.765e-05  y = 6.8051 (2)  else  y = 7.7963 (5)  else  if x1 <= 8.15e-05  y = 5.7805 (3)  else  y = 7.1516 (2)  else  if x1 <= 8.9e-05  if x3 <= 0.021697  if x4 <= 0.019216  if x1 <= 5.825e-05  y = 5.7125 (2)  else  y = 5.4101 (2)  else  if x1 <= 5.945e-05  y = 5.5617 (4)  else  y = 6.7675 (3)  else  y = 4.7933 (2)  else  if x3 <= 0.019768  if x3 <= 0.011813  y = 6.5261 (3)  else  if x3 <= 0.015865  y = 8.5623 (3)  else  y = 7.6049 (2)  else  if x1 <= 0.00056134  y = 6.3794 (8)  else  y = 6.9275 (2)  else  if x4 <= 0.029129  if x3 <= 0.02231  if x1 <= 0.002495  if x3 <= 0.018168  y = 9.9501 (5)  else  y = 9.5514 (2)  else  y = 11.43 (2)  else  y = 8.8014 (2)  else  if x3 <= 0.044073  y = 6.3023 (5)  else  y = 4.8767 (2)  else  if x1 <= 2.1e-05  y = 2.915 (2)  else  if x1 <= 0.0014431  y = 4.1847 (4)  else  y = 3.3293 (4)  else  if x2 <= 85.067  if x4 <= 0.020644  if x1 <= 0.0003028  if x2 <= 49.963  if x1 <= 3.68e-05  if x1 <= 1.87e-05  y = 2.8573 (3)  else  y = 3.8506 (2)  else  if x2 <= 28.773  if x3 <= 0.022736  if x4 <= 0.019428  y = 5.2396 (4)  else  y = 4.7705 (3)  else  if x1 <= 0.000144  y = 4.8028 (2)  else  if x3 <= 0.025149  y = 6.8179 (2)  else  y = 5.8478 (2)  else  if x4 <= 0.018118  if x1 <= 0.00014855  if x3 <= 0.018851  y = 4.8516 (4)  else  if x4 <= 0.016693  y = 3.7056 (2)  else  y = 4.2919 (2)  else  y = 5.1377 (3)  else  if x1 <= 0.00023155  y = 3.6112 (4)  else  y = 4.0912 (3)  else  if x3 <= 0.058276  if x4 <= 0.018955  y = 3.3216 (5)  else  y = 2.9518 (4)  else  y = 2.6767 (5)  else  if x2 <= 57.042  if x3 <= 0.026831  y = 8.1846 (2)  else  if x3 <= 0.033906  y = 6.8712 (3)  else  if x1 <= 0.00122  if x3 <= 0.048035  y = 6.0499 (4)  else  if x1 <= 0.00085  y = 5.0293 (2)  else  y = 5.6823 (3)  else  y = 6.3357 (9)  else  if x1 <= 0.0004419  if x1 <= 0.00037615  y = 4.3828 (2)  else  y = 3.5037 (2)  else  if x3 <= 0.066459  y = 5.5467 (7)  else  y = 4.6694 (4)  else  if x3 <= 0.05425  if x1 <= 0.0002783  if x1 <= 0.00015455  if x2 <= 49.96  if x2 <= 34.521  y = 4.0561 (10)  else  y = 4.6599 (4)  else  y = 3.6545 (2)  else  if x3 <= 0.046633  y = 3.3126 (4)  else  y = 3.7596 (2)  else  y = 4.9731 (15)  else  if x1 <= 0.00022422  if x3 <= 0.088293  if x4 <= 0.0302  y = 2.8694 (12)  else  y = 2.1676 (2)  else  y = 2.2299 (4)  else  if x4 <= 0.058231  if x2 <= 43.8  if x4 <= 0.031467  if x4 <= 0.027428  y = 5.2973 (2)  else  y = 4.6283 (3)  else  if x1 <= 0.012609  if x3 <= 0.073991  if x4 <= 0.038593  if x2 <= 32.436  y = 5.0881 (2)  else  y = 4.1571 (3)  else  y = 4.0225 (6)  else  if x4 <= 0.037919  y = 3.8336 (4)  else  y = 3.3436 (8)  else  y = 4.6234 (4)  else  if x4 <= 0.021582  y = 4.6425 (3)  else  if x2 <= 57.675  y = 3.5138 (2)  else  y = 2.8607 (6)  else  y = 2.6289 (8)  else  if x3 <= 0.19297  if x1 <= 0.0002915  if x2 <= 119.14  if x1 <= 3.45e-05  y = 1.7732 (3)  else  if x3 <= 0.11155  if x1 <= 0.000153  y = 2.5281 (2)  else  y = 3.0177 (2)  else  y = 1.9443 (2)  else  if x3 <= 0.12206  if x4 <= 0.013868  y = 1.9329 (2)  else  y = 2.1593 (7)  else  if x1 <= 0.00018595  y = 1.7185 (3)  else  y = 2.0374 (2)  else  if x4 <= 0.020199  if x1 <= 0.000878  if x3 <= 0.090036  y = 3.5976 (2)  else  if x4 <= 0.018439  y = 2.369 (6)  else  if x1 <= 0.0004394  y = 2.9121 (2)  else  y = 3.336 (2)  else  if x3 <= 0.099839  y = 4.023 (2)  else  if x1 <= 0.001264  y = 3.5244 (4)  else  y = 3.0681 (2)  else  if x1 <= 0.0010015  if x4 <= 0.021054  y = 2.5259 (3)  else  y = 2.1621 (5)  else  if x3 <= 0.1441  y = 2.8075 (2)  else  y = 2.5268 (2)  else  if x4 <= 0.022664  y = 2.0779 (5)  else  if x1 <= 0.0005412  y = 1.4683 (6)  else  y = 1.7005 (8) |
| --- |

**Table A2.** M5 rule tree (M5RT) structure

| Number of rules: 41  Number of original input variables used: x1 (*C_v_*), x2 (*D_gr_*), x3 (*d/R*) and x4 (*λ*)  The decision rules:  if x2 > 24.361 and x2 <= 85.067 and x4 > 0.020644 and x3 > 0.05425 and x1 > 0.00022422 and x4 <= 0.058231 and x2 <= 43.8 then y = 7.3343 +157.24*x1 -19.243*x3 -48.203*x4 (32)  if x2 > 24.361 and x2 <= 85.067 and x1 <= 0.00030635 and x3 <= 0.050982 and x3 <= 0.027705 and x1 <= 0.0001499 then y = 5.6425 +11736*x1 -106.2*x3 (19)  if x3 <= 0.035443 and x1 > 0.00037108 and x2 <= 13.63 and x4 <= 0.076089 and x4 <= 0.06631 and x4 <= 0.043532 then y = 12.546 +851.4*x1 -235.62*x3 (28)  if x3 > 0.031965 and x2 <= 85.067 and x1 <= 0.00030635 and x3 > 0.050982 and x3 <= 0.065879 then y = 2.9332 (17)  if x3 > 0.031965 and x2 > 85.067 and x3 > 0.19297 and x4 > 0.022664 then y = 1.469 +205.32*x1 (14)  if x3 > 0.031965 and x3 > 0.086324 and x1 <= 0.001041 and x1 <= 0.00044385 and x3 <= 0.1237 and x1 > 0.0002025 then y = 2.563 (11)  if x3 > 0.031965 and x2 > 85.067 and x1 > 0.0002965 and x4 <= 0.020195 then y = 4.3 -8.4101*x3 (15)  if x3 > 0.031965 and x2 <= 85.067 and x1 > 0.000285 and x4 > 0.054729 then y = 4.208 -15.607*x3 (13)  if x2 <= 43.8 and x3 <= 0.011102 and x1 > 8.4e-05 and x2 <= 10.751 then y = 10.239 (17)  if x2 <= 43.8 and x3 > 0.019183 and x1 > 7.29e-05 and x4 > 0.024609 and x3 > 0.039918 then y = 4.5317 (15)  if x2 <= 43.8 and x3 > 0.019183 and x1 > 8.1e-05 and x1 > 0.00029423 and x4 <= 0.045347 and x3 > 0.0233 and x3 > 0.032797 and x1 > 0.00122 then y = 6.4059 (11)  if x2 <= 40.156 and x3 > 0.019183 and x1 > 8.1e-05 and x3 <= 0.032797 and x4 > 0.017653 and x1 > 0.00029423 then y = 6.4904 (12)  if x2 <= 40.156 and x3 <= 0.023488 and x1 > 4.35e-06 and x3 <= 0.011398 and x1 > 1.19e-05 and x2 <= 18.284 and x1 <= 0.0001464 then y = 9.3121 -0.097928*x2 -132.63*x3 (22)  if x2 <= 40.156 and x3 <= 0.023488 and x1 > 4.35e-06 and x3 > 0.0093414 and x1 > 8.9e-05 and x3 <= 0.019183 then y = 8.6065 -21.68*x4 (13)  if x2 <= 40.156 and x3 <= 0.0093414 and x1 <= 1.285e-05 and x1 > 4.35e-06 then y = 6.31 (11)  if x3 <= 0.065239 and x3 > 0.0093414 and x2 > 40.156 and x1 > 0.000285 and x4 > 0.01798 then y = 11.67 +2282.9*x1 -0.083166*x2 +49.091*x3 -308.22*x4 (11)  if x2 > 40.156 and x2 <= 85.067 and x4 > 0.018084 and x4 <= 0.021558 and x1 > 8.275e-05 then y = 3.5467 +845.85*x1 (16)  if x3 > 0.065239 and x1 <= 0.0008625 and x2 > 84.628 and x1 > 0.00011265 then y = 2.1395 (20)  if x3 <= 0.065239 and x3 > 0.0093414 and x1 <= 1.92e-05 and x3 <= 0.019276 then y = 4.4446 (6)  if x3 <= 0.065239 and x3 > 0.023488 and x1 <= 8.915e-05 and x1 > 2.03e-05 then y = 4.4683 -0.016093*x2 (8)  if x3 <= 0.065239 and x3 > 0.010944 and x1 > 1.92e-05 and x3 > 0.030819 and x1 > 0.0002894 and x1 > 0.0007845 then y = 6.5029 -14.303*x3 (7)  if x2 > 40.156 and x4 <= 0.018617 and x1 > 9.45e-05 and x2 > 49.331 and x4 <= 0.017017 then y = 5.0253 (7)  if x2 > 40.156 and x3 > 0.060683 and x1 <= 0.001345 and x4 > 0.021974 and x4 <= 0.027504 and x2 <= 125.21 then y = 2.7419 (10)  if x2 > 49.331 and x2 > 85.067 then y = 2.1417 +786.96*x1 -3.6186*x3 (13)  if x2 <= 49.331 and x3 > 0.010944 and x1 > 1.84e-05 and x3 <= 0.044059 and x4 > 0.017553 and x2 <= 19.069 and x1 <= 5.41e-05 then y = 5.6568 (6)  if x2 <= 49.331 and x3 > 0.011422 and x1 > 2.17e-05 and x3 <= 0.030819 and x1 <= 0.00031028 and x2 <= 19.069 then y = 7.6513 -3605.2*x1 -22.1*x4 (6)  if x2 <= 49.331 and x3 > 0.011422 and x4 > 0.018954 and x1 > 7.575e-05 then y = 5.0209 (13)  if x3 <= 0.023488 and x1 > 8.45e-06 and x3 > 0.0091601 and x1 <= 0.00017785 then y = 5.6983 (5)  if x3 <= 0.023488 and x1 > 8.45e-06 and x3 <= 0.007696 and x1 <= 1.965e-05 then y = 5.3993 +1.6704e+05*x1 (6)  if x2 <= 49.331 and x1 > 1.845e-05 and x3 > 0.023862 then y = 7.9986 -55.447*x3 (8)  if x3 <= 0.023488 and x1 > 1.225e-05 and x3 > 0.007696 then y = 13.084 -284.66*x4 (5)  if x3 > 0.015258 and x4 <= 0.019147 and x1 <= 0.00029825 then y = 3.2111 (4)  if x3 <= 0.015258 and x1 <= 1.225e-05 and x4 > 0.0154 then y = 4.6835 (3)  if x3 <= 0.015258 and x1 > 1.205e-05 then y = 9.4814 (3)  if x4 > 0.018406 and x4 > 0.020814 and x4 > 0.02967 and x1 > 8.75e-05 then y = 2.1858 (3)  if x4 <= 0.018406 and x1 <= 0.00016485 then y = 5.3486 (2)  if x4 <= 0.018406 then y = 4.4223 (2)  if x4 <= 0.020814 then y = 2.2893 (2)  if x4 <= 0.022118 then y = 2.7468 (2)  if x1 <= 8.15e-05 then y = 2.915 (2)  y = 3.4969 (2) |
| --- |
